# Genistein Alleviates Diet-Induced Hypercholesterolemia via the Gut Microbiota–Bile Acid–SCFA Axis in Mice

**DOI:** 10.3390/foods15213986

**Published:** 2026-11-06

**Authors:** Na-Na Zhang, Yao-Sen Wang, Xin-Xin Guo, Jing Chen, Feng-Zhong Wang, Bei Fan

**Affiliations:** 1 Institute of Food Science and Technology, Chinese Academy of Agricultural Sciences/Key Laboratory of Agro-Products Processing, Ministry of Agriculture, Beijing 100193, China; 2 College of Food Science and Technology, Agricultural University of Hebei, Baoding 071001, China

**Keywords:** isoflavone, cholesterol-lowering effect, intestinal microflora, microbial metabolites

## Abstract

Hypercholesterolemia is a major risk factor for cardiovascular diseases. Genistein, a major bioactive isoflavone, has shown cholesterol-lowering potential, but the mechanisms underlying this effect remain unclear. In this study, genistein was purified from fermented soybean meal, and ICR mice fed a high-cholesterol diet were treated with genistein (40 mg/kg body weight/day) from fermented soybean meal for eight weeks. Genistein reduced body weight gain without affecting food intake, and decreased serum total cholesterol and low-density lipoprotein cholesterol. Genistein increased fecal total bile acid excretion and decreased serum total bile acids, accompanied by elevated fecal ursodeoxycholic acid and muricholic acid derivatives. Gut microbiota analysis showed restored alpha diversity, a decreased Firmicutes/Bacteroidetes ratio, and enriched *Akkermansia*, *Parabacteroides* and norank_f__Muribaculaceae. Fecal short-chain fatty acids (SCFAs) were increased, especially butyrate and valerate, and propionate was restored to levels comparable to normal controls. At the mRNA level, genistein treatment reduced intestinal farnesoid X receptor and fibroblast growth factor 15 expression and increased hepatic cholesterol 7α-hydroxylase expression, accompanied by altered expression of genes involved in cholesterol synthesis and efflux. Correlation analysis linked genistein-enriched taxa with improved cholesterol, bile acid, and SCFA profiles. In conclusion, purified genistein may alleviate diet-induced hypercholesterolemia by affecting the bile acid pool, gut microbiota, and SCFA production. These findings provide mechanistic insights into the cholesterol-lowering effects of genistein and may offer a scientific basis for the future development of genistein-based functional ingredients from soybean-derived materials.

## 1. Introduction

Hypercholesterolemia is a leading modifiable risk factor for cardiovascular diseases [1]. Elevated total cholesterol is estimated to cause approximately 2.6 million preventable deaths annually, accounting for 4% of all global deaths [2]. The 2026 ACC/AHA Multisociety Guideline on the Management of Dyslipidemia recommends dietary patterns that emphasize fruits, vegetables, nuts, legumes, whole grains, and fiber, while replacing saturated and trans fats with unsaturated fats, as the foundation of prevention and management [3]. However, dietary adherence is constrained by cost, access, and patient preference, with adherence rates of only 16% for secondary and 7% for primary prevention [2]. Moreover, statin use can be accompanied by adverse effects including abnormal liver enzymes and rhabdomyolysis [4]. Consequently, food-derived bioactive compounds from sustainable plant sources, particularly those intrinsic to recommended food groups such as legumes, have attracted increasing attention as complementary strategies for cholesterol management.

Genistein is one of the major bioactive isoflavones in soybeans and soy-derived products [5]. In soybean meal, isoflavones predominantly exist as glycosylated forms with limited bioavailability. Microbial fermentation can convert these glycosides into their corresponding aglycones, thereby increasing the genistein content [6,7]. Animal and cell studies have shown that genistein can lower serum total cholesterol and low-density lipoprotein cholesterol by upregulating hepatic low-density lipoprotein receptor, inhibiting sterol regulatory element-binding protein-2 (SREBP-2) and 3-hydroxy-3-methylglutaryl-coenzyme A reductase (HMGCR) expression, and promoting cholesterol 7α-hydroxylase (CYP7A1)-mediated bile acid synthesis [8,9]. However, the absolute bioavailability of genistein remains low, and a considerable proportion of unabsorbed parent or conjugated metabolites enter the colon, where they undergo hydrolysis and reduction by gut microbiota [10,11]. This suggests that gut microbiota may be an important target or mediator for its cholesterol-regulating effects.

In recent years, the mechanism by which gut microbiota regulates cholesterol homeostasis through bile acid and short-chain fatty acid (SCFA) metabolism has attracted increasing attention. The liver synthesizes primary bile acids from cholesterol, which are excreted into the intestine and converted by microbial bile salt hydrolase and 7α-dehydroxylase into secondary bile acids [12]. In addition to participating in lipid digestion, bile acids act as signaling molecules to activate FXR, thereby regulating hepatic CYP7A1 expression to affect cholesterol conversion and absorption [13]. Meanwhile, SCFAs, as major microbial metabolites, have been increasingly recognized for their roles in modulating cholesterol metabolism and FXR signaling, either by regulating hepatic cholesterol synthesis and bile acid synthesis or by acting as indirect modulators of FXR [14]. Therefore, changes in microbiota can indirectly regulate cholesterol metabolism by altering the size, composition, and signaling strength of the bile acid pool, as well as SCFA production. Studies have shown that genistein can alter gut microbiota structure and fecal/serum bile acid profiles, such as increasing SCFA-producing bacteria, regulating bile salt hydrolase-related bacteria, and changing the ratio of primary to secondary bile acids [15,16]. These findings led us to hypothesize that genistein, a dietary isoflavone present in fermented soybean meal, would alleviate high-cholesterol diet-induced hypercholesterolemia in mice by modulating the gut microbiota–bile acid–SCFA axis.

To test this hypothesis, this study used purified genistein, isolated from fermented soybean meal, as a defined bioactive isoflavone and aimed to investigate its cholesterol-lowering effect and the associated changes in gut microbiota, bile acids, and SCFAs. Using 16S rRNA gene sequencing, targeted bile acid metabolomics, SCFA analysis, and qPCR-based gene expression analysis, we analyzed the regulatory effects of genistein on gut microbiota composition, bile acid profiles, SCFA production, and the expression of genes related to FXR–FGF15 signaling, bile acid synthesis, and cholesterol homeostasis. We further explored whether these changes were associated with the cholesterol-lowering effect of genistein, particularly in relation to the gut microbiota–bile acid–SCFA axis. This study provides experimental evidence and potential mechanistic insights into the cholesterol-lowering effects of purified genistein and may support the development of genistein-based functional ingredients from soybean-derived materials.

## 2. Materials and Methods

### 2.1. Reagents and Raw Materials

TRIzol reagents were from Invitrogen (Carlsbad, CA, USA). GoScript reverse transcription (RT) system and Power SYBR Green PCR master mix were from Promega (Madison, WI, USA). Total cholesterol (TC), triglyceride (TG), high-density lipoprotein cholesterol (HDL-C), and low-density lipoprotein cholesterol (LDL-C) were purchased from Nanjing JianCheng Bioengineering Institute (Nanjing, China). Total bile acid (BA) assay kits were purchased from Beyotime Biotechnology (Shanghai, China).

### 2.2. Fermentation, Extraction, Separation, and Identification of Genistein from Soybean Meal

Soybean meal was fermented with Bacillus subtilis according to a previously described method with slight modifications [17]. Briefly, the activated culture was centrifuged (4000 rpm, 4 °C, 10 min) to collect the bacterial cells, which were then resuspended in saline to prepare an inoculum at a concentration of 25 mg/mL, with a final bacterial concentration of 10^7^ CFU/mL. Then, 1.8 mL of this suspension was inoculated into a medium containing soybean meal (15 g) and water (60 mL). Fermentation was carried out at 37 °C with shaking at 200 rpm for 3 days. The fermented soybean meal was freeze-dried and extracted with 80% aqueous methanol (1:10, *w*/*v*) at room temperature for 24 h with stirring, followed by centrifugation (15,000 rpm, 10 min). The supernatant was evaporated, fractionated with water and ethyl acetate, and purified by preparative HPLC using a C18 column with methanol/10 mM ammonium acetate buffer (60:40, *v*/*v*) at 254 nm. The genistein peak (retention time, 71 min) was collected, and its identity was confirmed by HPLC against a standard; the obtained genistein had a purity of 98%. This procedure was adapted from previously reported methods with minor modifications [18]. Analytical HPLC and TLC profiles of the final product are shown in Figure S1.

### 2.3. Animals and Treatment

Thirty-six 5-week-old male Institute of Cancer Research (ICR) mice were obtained from Vital River Co., Ltd. (Beijing, China). Animals were housed six per cage under a 12-h light/12-h dark cycle at 23 ± 2 °C and 50% ± 10% humidity. Standard chow and water were provided ad libitum. All experimental procedures complied with the National Research Council’s Guide for the Care and Use of Laboratory Animals and were approved by the Animal Care and Use Committee of Inner Mongolia Medical University (Permit No. YKD202401208; Approval Date: 9 March 2024).

After 1 week of acclimatization, mice were randomly allocated into three groups: control (C), model (M), and genistein (GEN). The diets were commercially custom-prepared by Trophic Animal Feed High-tech Co., Ltd. (Nantong, China). Its complete composition, including cholesterol concentration and major components, is provided in Supplementary Table S1. The C group received normal feed, while the M and GEN groups were fed a high-cholesterol diet (1.25% cholesterol and 0.5% bile salt). In addition, the GEN group received GEN solution (40 mg/kg body weight/day) by gavage. The genistein dose was based on a previous study [19], in which the intermediate dose was effective and well tolerated; this dose was therefore chosen to balance efficacy and safety. The C and M groups received an equal volume of normal saline by gavage. Gavage administration continued for 8 weeks. Food intake and body weight were recorded weekly. During the last week of the 8-week intervention, fresh fecal samples were collected for 16S rRNA gene sequencing, targeted bile acid metabolomics, SCFA analysis, and total bile acid measurement. At the end of week 8, mice were anesthetized with isoflurane in an anesthesia chamber and euthanized by cardiac exsanguination. Blood, liver, and ileum samples were collected for subsequent biochemical analyses.

### 2.4. Analysis of Serum and Liver Lipid Profiles

Serum TC, TG, HDL-C, and LDL-C, as well as hepatic TC and TG, were measured using commercial enzymatic assay kits (Nanjing Jiancheng Bioengineering Institute, Nanjing, China) according to the manufacturer’s instructions.

### 2.5. Analysis of Total Bile Acid in Serum and Feces

Total bile acids in serum and feces were measured using the same commercial bile acid assay kit (Beyotime, Shanghai, China) based on the 3-α-hydroxysteroid dehydrogenase method. Blood samples were centrifuged to separate serum. Liver tissue and fecal samples were homogenized and centrifuged, and the supernatant was collected. All supernatants were then subjected to the same enzymatic assay according to the manufacturer’s instructions.

### 2.6. 16S rRNA Gene Sequencing

16S rRNA gene sequencing was performed as previously described [20]. Total DNA was extracted from cecal contents using the QIAamp DNA Stool Mini Kit (Qiagen, Valencia, CA, USA) according to the manufacturer’s instructions. DNA integrity was examined by 1% agarose gel electrophoresis. The V3–V4 hypervariable region of the bacterial 16S rRNA gene was amplified on an ABI GeneAmp^®^ 9700 PCR System (Applied Biosystems, Carlsbad, CA, USA). The amplicons were quantified using a QuantiFluor™ ST Handheld Fluorometer with UV/Blue channels (Promega Corporation, Madison, WI, USA). Sequencing was performed on an Illumina MiSeq platform at Majorbio Bio-Pharm Technology Co., Ltd. (Shanghai, China). Raw reads were quality-filtered and screened to remove low-quality sequences, adaptors, and chimeras. The remaining high-quality sequences were clustered into operational taxonomic units (OTUs) or denoised into amplicon sequence variants, followed by taxonomic annotation against the SILVA reference database. To minimize the effects of sequencing depth on alpha and beta diversity measures, the number of 16S rRNA gene sequences per sample was rarefied to 23,264. Alpha diversity was assessed using the Shannon index (community diversity) and the Chao index (community richness). Differences in alpha diversity between groups were evaluated using the Kruskal–Wallis test followed by Dunn’s post hoc test. Beta diversity (β-diversity) was evaluated based on Bray–Curtis distances. Principal coordinate analysis (PCoA) was used for visualization. Differences in microbial community composition among groups were tested using ANOSIM. The relative abundances of bacterial taxa were summarized at the phylum and genus levels.

### 2.7. Bile Acids (BAs) and Targeted Short Chain Fatty Acids (SCFAs) Analysis

Bile acids and short-chain fatty acids (SCFAs) in fecal samples were quantified as described previously [20]. Briefly, 20 mg of fecal samples were suspended in 800 μL of 0.5% phosphoric acid aqueous solution, homogenized, and centrifuged at 8000 rpm for 10 min. The extract was filtered through a 0.22-μm sterile membrane, transferred to a chromatographic vial, and analyzed by GC-MS (Agilent Technologies, Santa Clara, CA, USA) for SCFAs. Bile acids in fecal samples were measured by ultra-performance liquid chromatography–tandem mass spectrometry (UPLC-MS/MS) (AB Sciex, Framingham, MA, USA). A 25 mg amount of the fecal samples was diluted with 380 μL of methanol–water (4:1, *v*/*v*), mixed with deuterated internal standards, homogenized, and centrifuged at 20,000× *g* for 10 min. The supernatant was dried and reconstituted in methanol–water (1:1). After filtration through a 0.22-μm PVDF membrane, samples were analyzed by UPLC-MS/MS.

### 2.8. Reverse Transcription (RT)-qPCR

Total RNA was extracted from liver and ileum tissues using TRIzol reagent (Invitrogen, Carlsbad, CA, USA) according to the manufacturer’s instructions. Complementary DNA (cDNA) was synthesized using GoScript reverse transcription (RT) system (Promega, Madison, WI, USA) and random primers. Gene expression was measured with Power SYBR Green PCR Master Mix (Promega, Madison, WI, USA) on an ABI 7500 real-time PCR system (Applied Biosystems, Foster City, CA, USA). Relative expression was calculated using the ΔΔCt method, with the model group as the calibrator and GAPDH as the internal reference gene. Primer sequences are listed in Table S2.

### 2.9. Statistical Analysis

Sample size was determined a priori for the primary outcome (serum TC) using G*Power 3.1.9.7 (one-way ANOVA, three groups, α = 0.05, power = 0.80). Based on our pilot data (group means 5.023, 6.587, and 5.693 mmol/L; pooled SD = 0.640 mmol/L; Cohen’s *f* = 1.00, calculated using the “Effect size from means” function), the required minimum was 4 mice per group. We used *n* = 6 per group to cover technical attrition and ensure adequate precision across secondary endpoints. Serum TC was the prespecified primary outcome; all other endpoints were secondary/exploratory and were not used for sample size calculation. Statistical analyses were performed using GraphPad Prism version 9.0 (GraphPad Software Inc., La Jolla, CA, USA). For parametric data, one-way ANOVA followed by Dunnett’s test (equal variances) or Welch’s ANOVA followed by Dunnett’s T3 test (unequal variances) was used for comparisons versus the model group. For non-parametric data, Kruskal–Wallis followed by Dunn’s test was used. Two-way repeated measures ANOVA was used when time was considered as a variable. Only comparisons versus the model group were made; Control vs. Genistein was not compared because of different diets. MD with 95% CI are reported for comparisons versus the model group. Spearman correlation analysis was performed using an online tool (https://cloud.majorbio.com/page/tools/) (accessed on 3 September 2026) to assess correlations (coefficients range from −1.0 to +1.0) among gut microbiota, metabolites, and physiological and biochemical indices. Data are presented as mean ± SD. A value of *p* < 0.05 was considered statistically significant.

## 3. Results

### 3.1. Effect of GEN Treatment on Body Weight and Serum Lipid Levels in Mice

**Figure 1:**
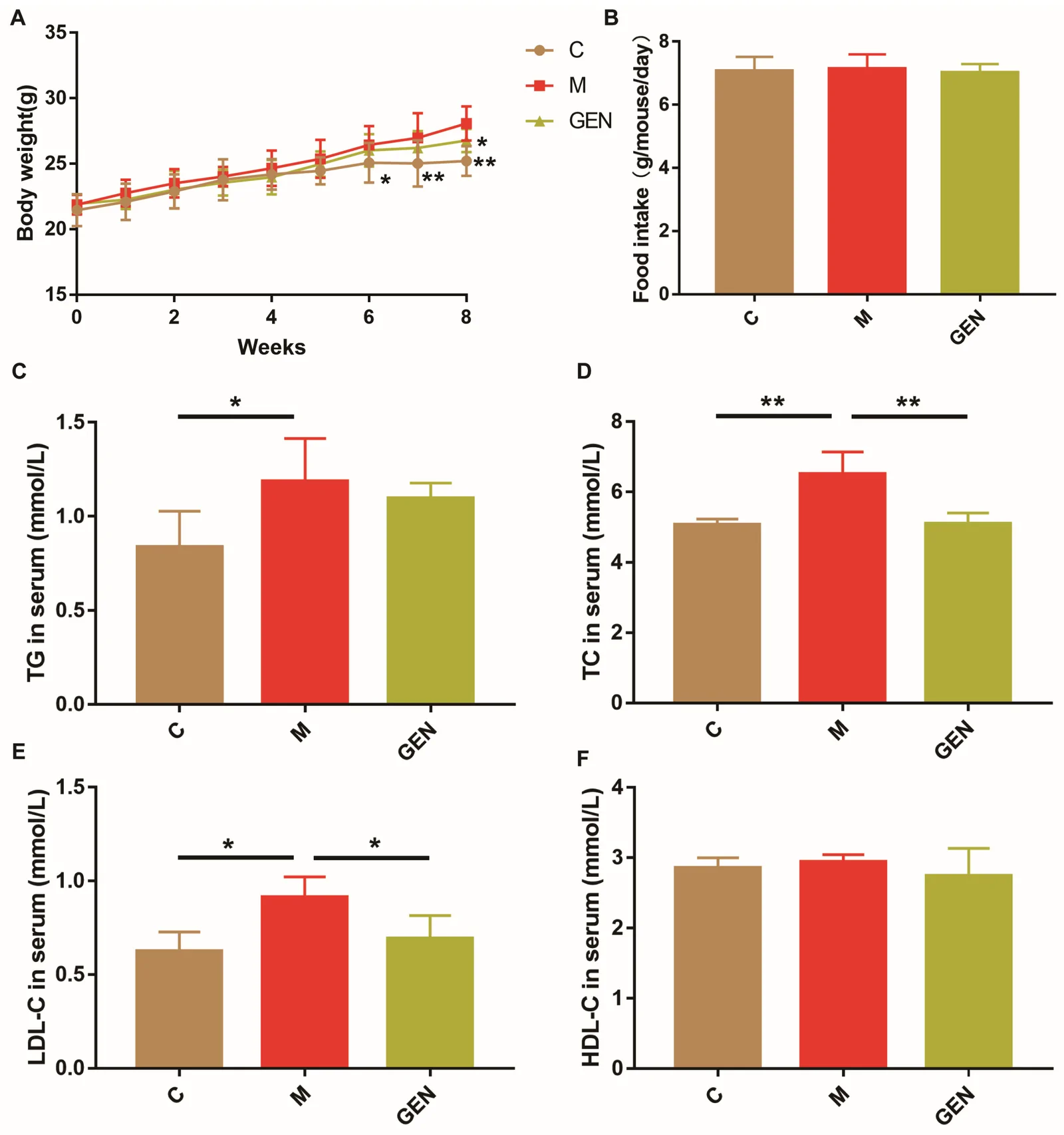
Effect of GEN treatment on body weight and serum lipid levels in mice. (**A**) Body weight; (**B**) Food intake; (**C**) Serum TG level; (**D**) Serum TC level; (**E**) Serum LDL-C level; (**F**) Serum HDL-C level. The data are presented as means ± SD; *n* = 6 mice/group. Two-way repeated measures ANOVA (**A**) and one-way ANOVA followed by Dunnett’s test (**B**–**F**) were used. * *p* value for vs. M < 0.05, ** *p* value for vs. M < 0.01.

Mice fed a high-cholesterol diet were administered 40 mg/kg body weight/day genistein for eight consecutive weeks to investigate the effects of genistein against diet-induced hypercholesterolemia. At the end of the experiment, body weight in the M group was significantly higher than in the C group (28.15 ± 1.28 vs. 25.52 ± 1.08 g; MD = 2.63 g, 95% CI 1.13 to 4.13; *p* < 0.01). Genistein administration significantly reduced body weight relative to the M group (26.47 ± 0.77 vs. 28.15 ± 1.28 g; MD = 1.68 g, 95% CI 0.18 to 3.18; *p* < 0.05) (Figure 1A), and these results were not related to changes in food intake (Figure 1B). Triglyceride (TG) levels were not significantly decreased in the GEN group compared with the M group (MD = 0.09 mM, 95% CI −0.23 to 0.41) (Figure 1C). Total cholesterol (TC) was markedly higher in the M group (6.50 ± 0.63 mmol/L) than in the C group (5.07 ± 0.16 mmol/L) (MD = 1.43 mmol/L, 95% CI 0.66 to 2.21; *p* < 0.01) (Figure 1D). Supplementation with GEN significantly decreased TC (MD = 1.42 mmol/L, 95% CI 0.68 to 2.16; *p* < 0.01) (Figure 1D). Low-density lipoprotein cholesterol (LDL-C) was significantly higher in the M group (0.91 ± 0.11 mmol/L) than in the C group (0.62 ± 0.10 mmol/L) (MD = 0.28 mmol/L, 95% CI 0.12 to 0.44; *p* < 0.05) (Figure 1E). The GEN group exhibited significantly lower LDL-C than the M group (MD = 0.22 mmol/L, 95% CI 0.02 to 0.42; *p* < 0.05) (Figure 1E). No significant difference in high-density lipoprotein cholesterol (HDL-C) was observed between the M and GEN groups (MD = 0.20 mmol/L, 95% CI −0.26 to 0.66) (Figure 1F). These results suggested that genistein exerted a significant protective effect against diet-induced body weight gain and hypercholesterolemia.

### 3.2. Effect of GEN Treatment on Liver Lipid Metabolism and Total Bile Acid in Mice

**Figure 2:**
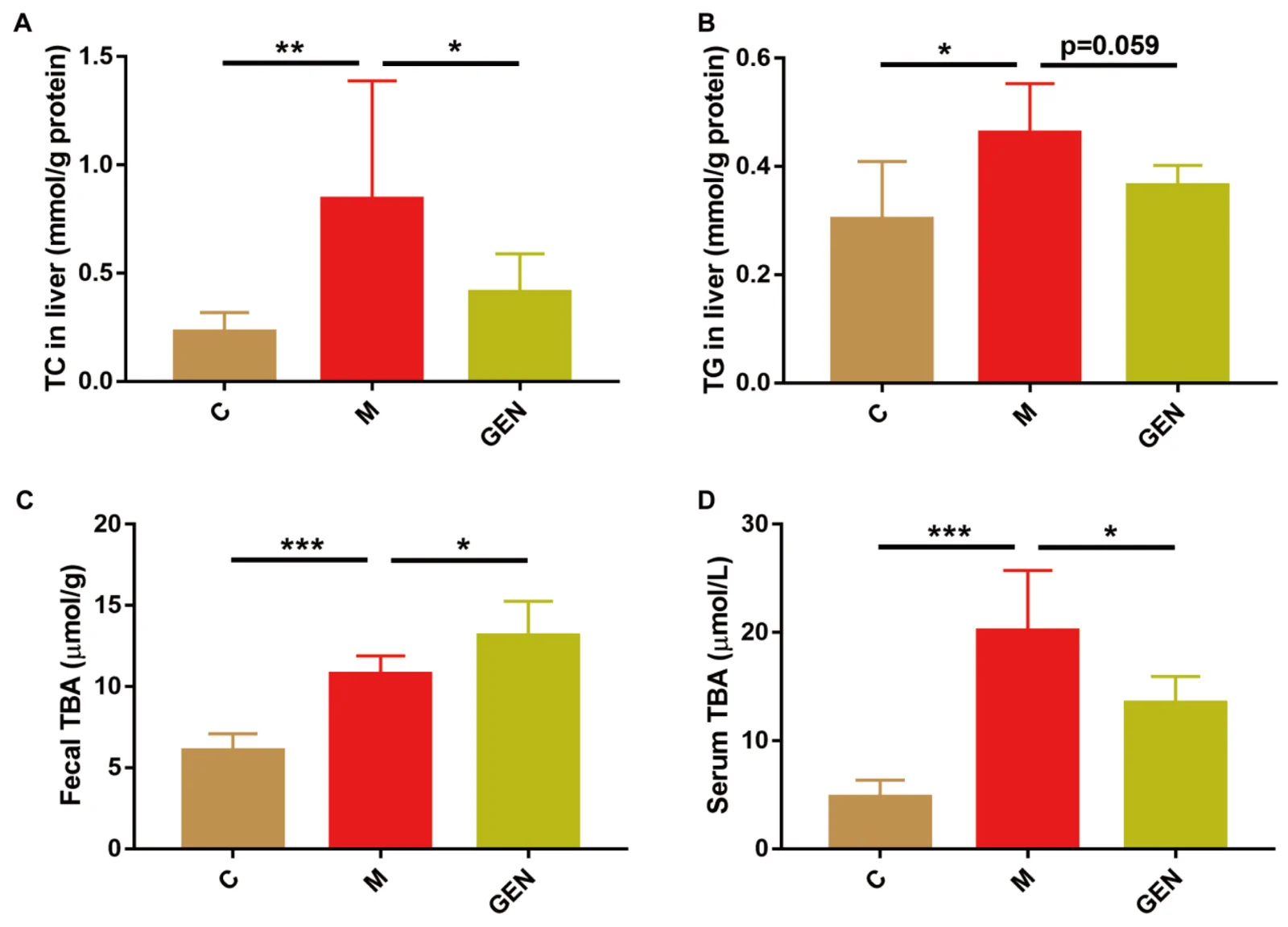
Effect of GEN treatment on liver lipid metabolism and total bile acid in mice. (**A**) TC level in liver; (**B**) TG level in liver; (**C**) Fecal TBA level; (**D**) Serum TBA level. The data are presented as means ± SD; *n* = 6 mice/group. Welch’s ANOVA followed by Dunnett’s T3 test (**A**–**C**) and one-way ANOVA followed by Dunnett’s test (**D**). * *p* value for vs. M < 0.05, ** *p* value for vs. M < 0.01, *** *p* value for vs. M < 0.001.

Next, we investigated the regulatory effects of GEN on lipid metabolism in the liver. As shown in Figure 2A,B, hepatic TC contents in the M group (0.94 ± 0.39 mmol/g protein) were significantly higher than those in the C group (0.24 ± 0.07 mmol/g protein) (MD = 0.69 mmol/g protein, 95% CI 0.25 to 1.14; *p* < 0.01). Hepatic TC was significantly lower in GEN mice than in the M group (MD = 0.51 mmol/g protein, 95% CI 0.08 to 0.95; *p* < 0.05) (Figure 2A). However, hepatic TG did not differ significantly between the M and GEN groups (MD = 0.10 mmol/g protein, 95% CI −0.01 to 0.21) (Figure 2B). To investigate bile acid metabolism in mice, total bile acids (TBAs) were quantified in serum and feces, with the results presented in Figure 2C,D. Compared with the C group, the total bile acids in serum and feces of the M group were increased to 20.17 ± 5.56 μmol/L and 10.77 ± 0.99 μmol/g, respectively. Compared with the M group, GEN administration significantly increased fecal TBA (MD = −2.35 μmol/g, 95% CI −4.64 to −0.05; *p* < 0.05). By contrast, serum TBA levels were significantly decreased after GEN administration (MD = 6.66 μmol/L, 95% CI 1.56 to 11.77; *p* < 0.05). Collectively, these findings suggest that genistein may increase fecal bile acid excretion and influence bile acid metabolism in mice.

### 3.3. Genistein Modulates the Composition of Gut Microbiota in Mice

**Figure 3:**
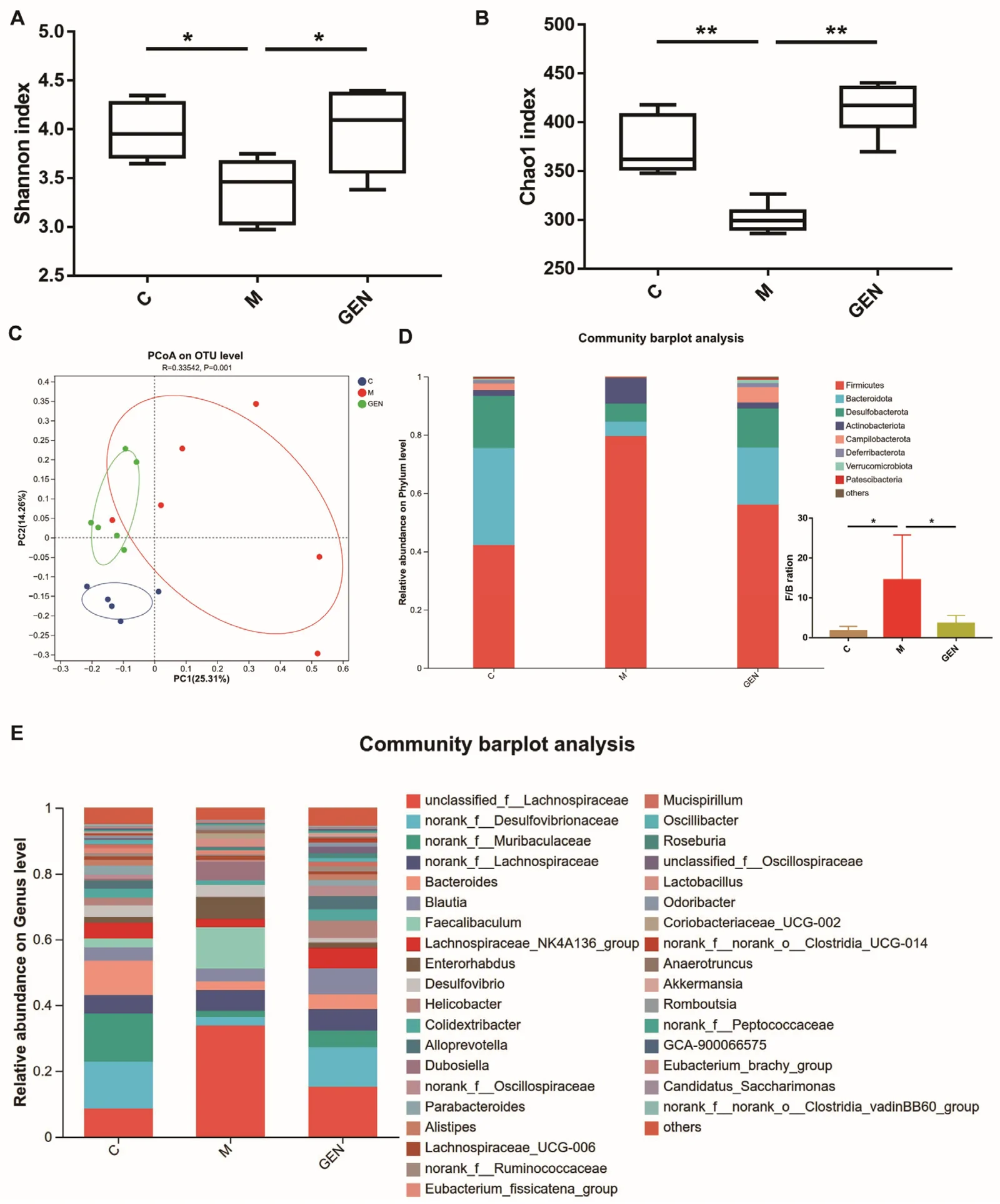
Genistein modulates the composition of gut microbiota in mice. (**A**) Shannon index; (**B**) Chao index; (**C**) Gut microbiome β-diversity; (**D**) Relative abundance of gut microbiome in phylum level; (**E**) Relative abundance of gut microbiome in genus level. *n* = 6 mice/group. For Shannon, Chao and F/B, one-way ANOVA followed by Dunnett’s test was used. * *p* value for vs. M < 0.05, ** *p* value for vs. M < 0.01.

Given that the gut microbiota is known to modulate cholesterol homeostasis and bile acid metabolism, this study aimed to assess global alterations in the gut microbial community following genistein treatment. For this purpose, the 16S rRNA gene sequences of fecal microbial samples collected from mice in the C, M and GEN groups were analyzed. As illustrated in Figure 3A,B, the Shannon and Chao1 indexes were markedly reduced in the M group relative to the C group, suggesting that the high-cholesterol diet induced alterations in gut microbiota abundance. Notably, these effects were partially restored by genistein administration compared with M group (Shannon index: MD = −0.60, 95% CI −1.10 to −0.09; *p* < 0.05; Chao1 index: MD = −112.9, 95% CI −146.2 to −79.6; *p* < 0.001) (Figure 3A,B). Principal coordinate analysis (PCoA) revealed that genistein administration altered the composition of the intestinal microbiota (ANOSIM: R = 0.33, *p* = 0.001) (Figure 3C). At the phylum level, genistein treatment resulted in a decrease in Firmicutes and an increase in Bacteroidetes, leading to a reduced Firmicutes/Bacteroidetes ratio (F/B) compared with the M group (MD = 1.93, 95% CI 1.32 to 2.55; *p* < 0.05) (Figure 3D). Genistein treatment also decreased the abundance of Actinobacteriota while increasing the abundances of Desulfobacterota and Campylobacterota (Figure 3D). At the genus level, the relative abundances of norank_f__Desulfovibrionaceae, norank_f__Muribaculaceae, Lachnospiraceae_NK4A136_group, *Alloprevotella*, *Akkermansia*, and *Parabacteroides* were significantly elevated in the GEN group relative to the M group while unclassified_f__Lachnospiraceae and *Dubosiella* were significantly reduced (Figure 3E). These data indicate that genistein treatment reshaped the gut microbial community, leading to alterations in overall community structure and in the relative abundances of several bacterial groups.

### 3.4. Genistein Reshapes Intestinal Bile Acid and Short-Chain Fatty Acid Profiles in Mice

**Figure 4:**
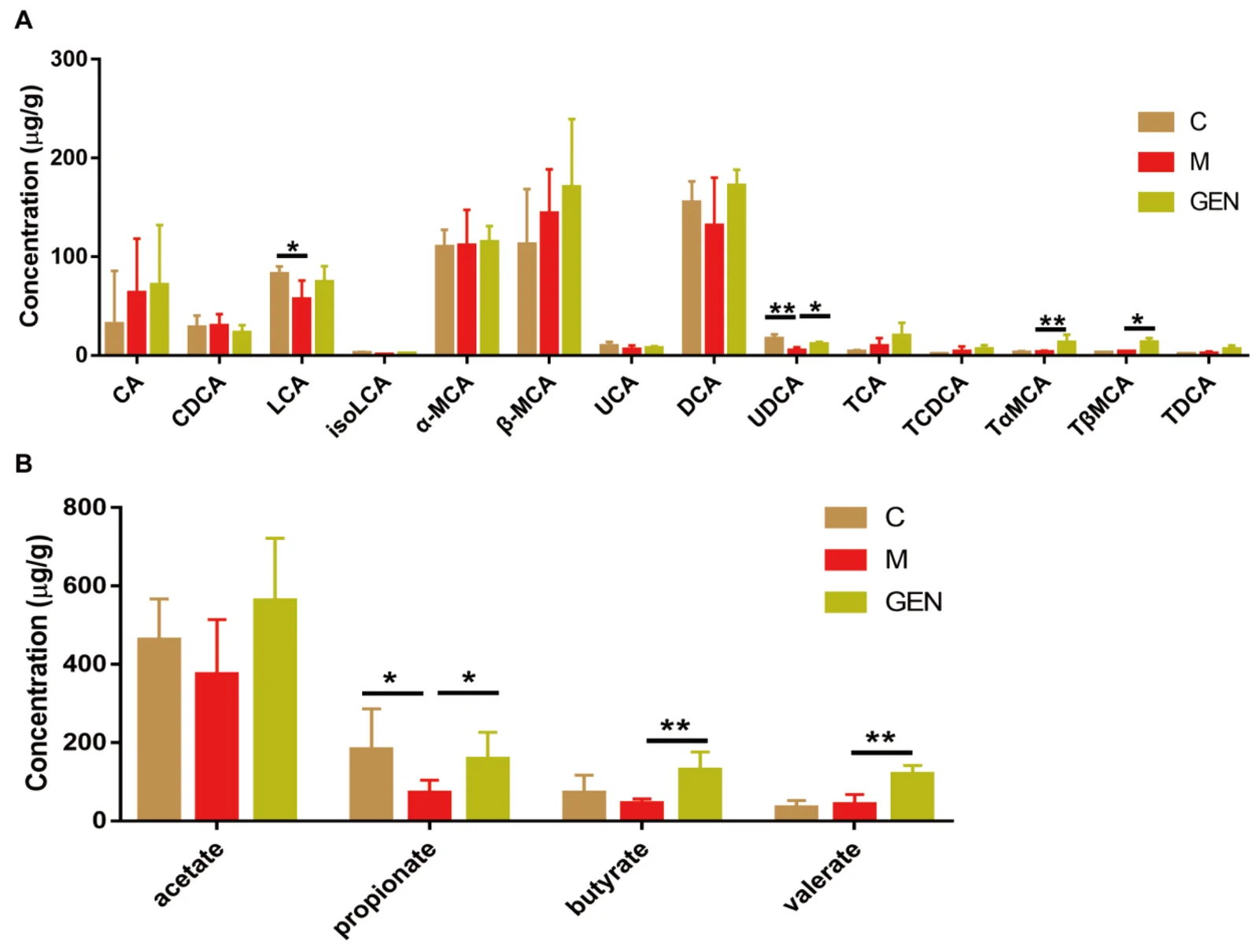
Genistein reshapes intestinal bile acid and short-chain fatty acid profiles in mice. (**A**) Fecal bile acid quantity; (**B**) Fecal SCFA quantity. The data are presented as means ± SD; Bile acid profiling: n = 6 mice/group; SCFA quantification: C *n* = 5, M *n* = 6, GEN *n* = 6; * *p* < 0.05, ** *p* < 0.01. For CA, UCA, and TCA, the Kruskal–Wallis test followed by Dunn’s test was used. For TβMCA, Welch’s ANOVA followed by Dunnett’s T3 test was used. For the other BAs and SCFAs, one-way ANOVA followed by Dunnett’s test was used. * *p* value for vs. M < 0.05, ** *p* value for vs. M < 0.01.

Given that bile acids (BAs) and short-chain fatty acids (SCFAs) are well-established regulators of cholesterol homeostasis, we next sought to evaluate whether genistein treatment altered their profiles in mice. The results showed that the fecal ursodeoxycholic acid (UDCA) content in the GEN group (11.43 ± 2.29 μg/g) was significantly higher than that in the M group (4.73 ± 3.74 μg/g; MD = −6.56 μg/g, 95% CI −11.77 to −1.36; *p* < 0.05; Figure 4A). The fecal abundances of tauro-α-muricholic acid (TαMCA) and TβMCA were elevated by 234.38% and 301.76% compared with the M group, respectively (TαMCA: MD = −9.71 μg/g, 95% CI −16.65 to −2.77; *p* < 0.01; TβMCA: MD = −10.06 μg/g, 95% CI −17.58 to −2.54; *p* < 0.05; Figure 4A). Compared with the M group, fecal SCFA analysis revealed elevated levels of SCFAs. Genistein-treated mice showed robust elevations in butyrate and valerate levels compared with M mice (butyrate: MD = −85.36 μg/g, 95% CI −148.1 to −22.60; *p* < 0.01; valerate: MD = −116.4 μg/g, 95% CI −179.4 to −53.44; *p* < 0.01; Figure 4B). GEN treatment significantly increased propionate compared with the M group (MD = −90.72 μg/g, 95% CI −180.7 to −0.72; *p* < 0.05; Figure 4B). These results suggest that genistein reshaped the intestinal bile acid pool and had a beneficial effect on increasing SCFA levels.

### 3.5. Genistein Alters the mRNA Expression of Markers of Cholesterol Metabolism in Mice

**Figure 5:**
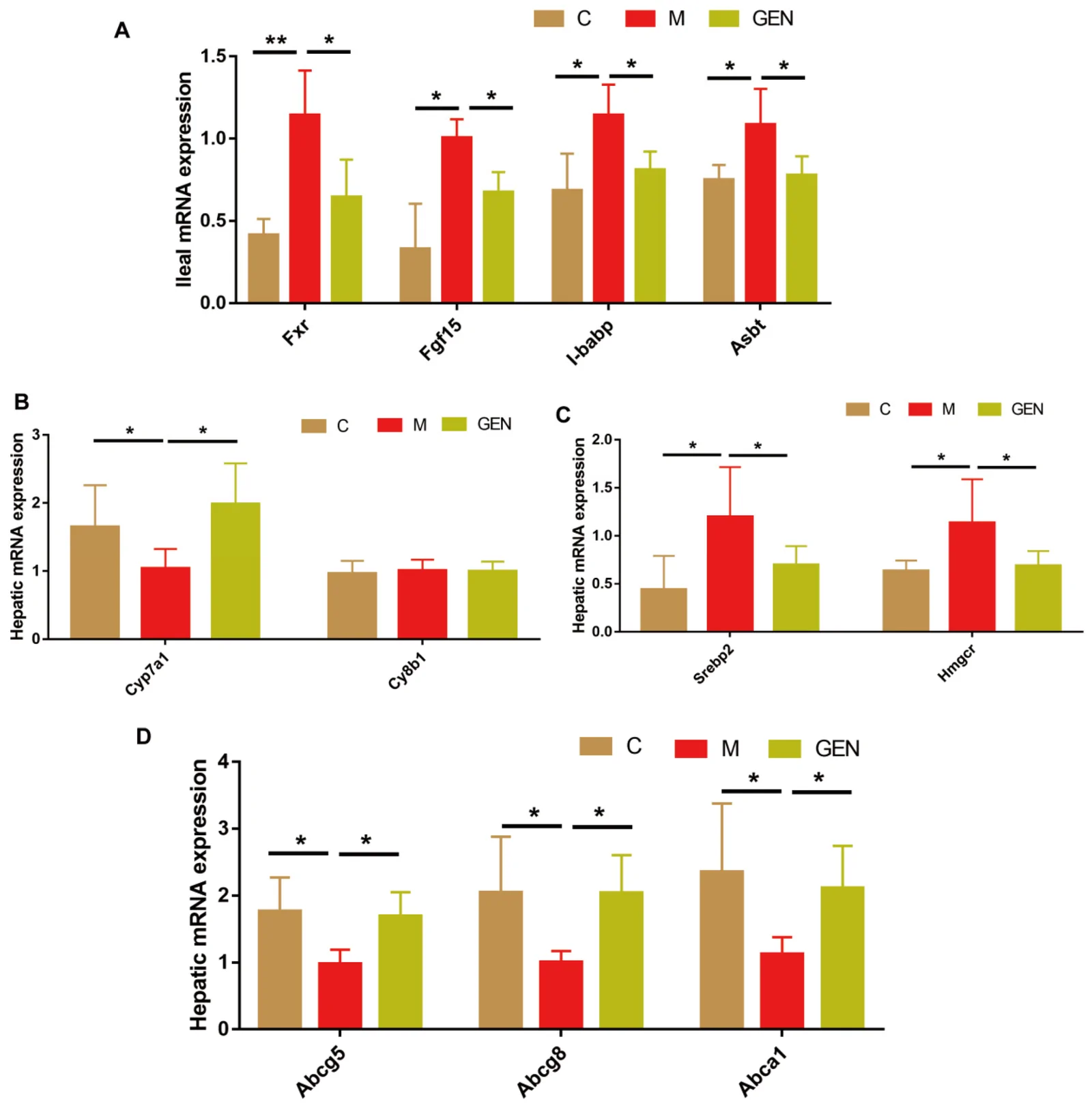
Genistein alters the mRNA expression of markers of cholesterol metabolism in mice. (**A**) Gene expression of *Fxr*, *Fgf15*, *Ibabp* and *Asbt*; (**B**) Gene expression of *Cyp7a1* and *Cyp8b1*; (**C**) Gene expression of *Srebp2* and *Hmgcr*; (**D**) Gene expression of *Abcg5*, *Abcg8* and *Abca1*. The data are presented as means ± SEM; *n* = 6 mice/group. For *Fgf15* and *Abca1*, Welch’s ANOVA followed by Dunnett’s T3 test was used. For the other genes, one-way ANOVA followed by Dunnett’s multiple comparisons test was used. * *p* value for vs. M < 0.05, ** *p* value for vs. M < 0.01.

Given the observed alterations in fecal bile acid and SCFA levels, we further investigated the molecular mechanisms underlying the regulation of cholesterol metabolism. Genistein administration reduced the farnesoid X receptor (*Fxr*) mRNA expression in the mouse distal ileum (MD = 0.50, 95% CI 0.10 to 0.89; *p* < 0.05; Figure 5A). Consistently, the mRNA levels of several genes associated with the FXR pathway, including fibroblast growth factor 15 (*Fgf15*), intestinal bile acid-binding protein (*Ibabp*), and apical sodium-dependent bile acid transporter (*Asbt*), were significantly lower in genistein-treated mice (*Fgf15*: MD = 0.36, 95% CI 0.01 to 0.71; *Ibabp*: MD = 0.35, 95% CI 0.03 to 0.68; *Asbt*: MD = 0.30, 95% CI 0.02 to 0.58; *p* < 0.05 for all; Figure 5A). We next examined the impact of genistein on hepatic bile acid and cholesterol metabolism. RT-qPCR analysis showed that hepatic expression of *Cyp7a1* was elevated in genistein-treated mice (MD = −0.94, 95% CI −1.77 to −0.11; *p* < 0.05; Figure 5B). Hepatic mRNA expression of genes involved in cholesterol synthesis, including *Srebp2* and *Hmgcr*, was reduced in genistein-treated mice (*Srebp2*: MD = 0.60, 95% CI 0.02 to 1.19; *Hmgcr*: MD = 0.50, 95% CI 0.04 to 0.96; *p* < 0.05 for all; Figure 5C). Notably, hepatic mRNA expression of ATP-binding cassette transporter G5/G8 (*Abcg5* and *Abcg8*) and ATP-binding cassette subfamily A member 1 (*Abca1*) was also elevated following genistein treatment (*Abcg5*: MD = −0.71, 95% CI −1.41 to −0.01; *Abcg8*: MD = −1.03, 95% CI −1.96 to −0.09; *Abca1*: MD = −1.12, 95% CI −2.21 to −0.03; *p* < 0.05 for all; Figure 5D). Taken together, these transcriptional changes suggest that genistein may modulate hepatic bile acid and cholesterol metabolism, potentially through FXR–FGF15 signaling.

### 3.6. Integrated Correlation Analysis Among Gut Microbiota, Metabolites, and Host Metabolic Markers

**Figure 6:**
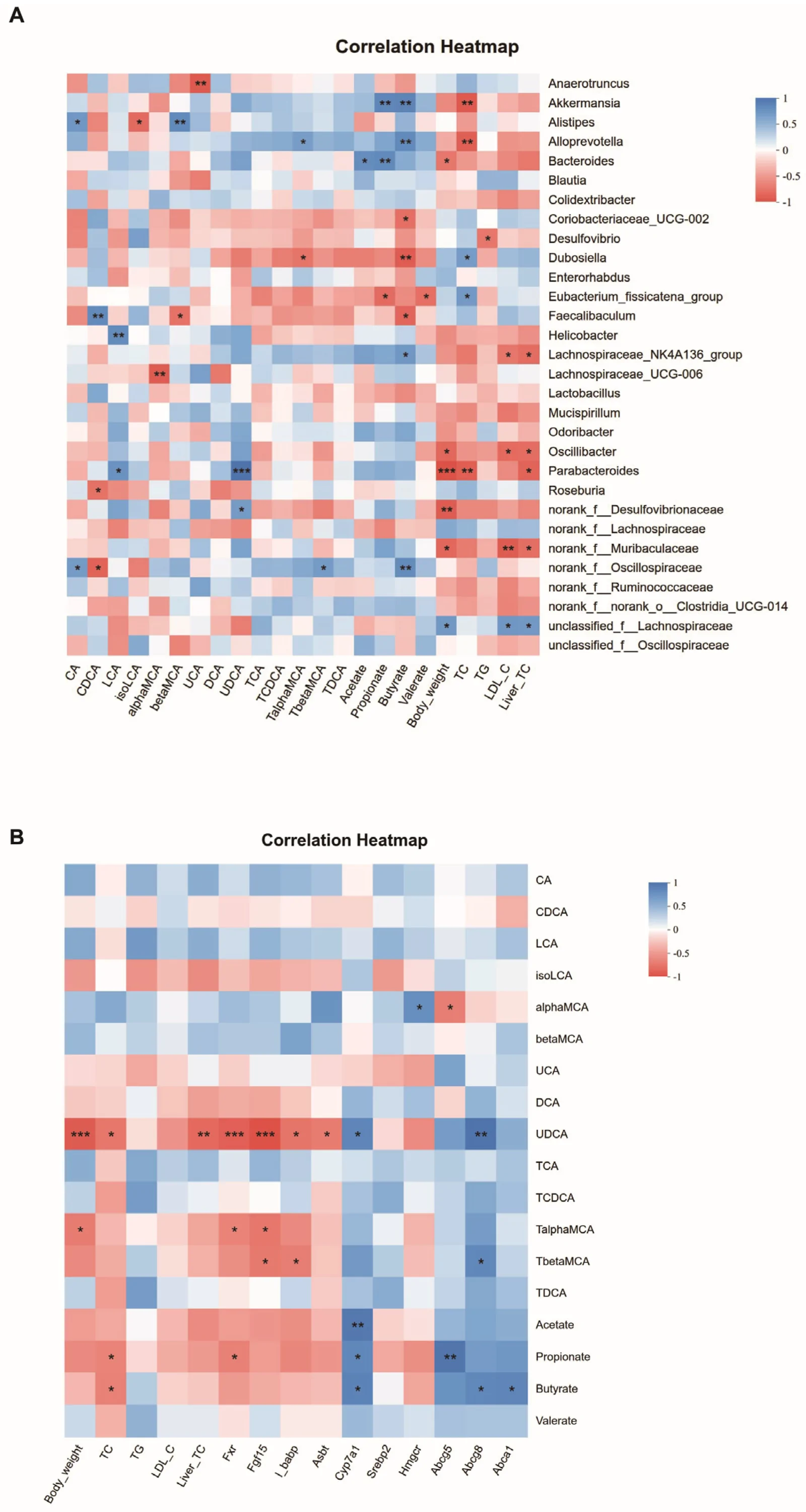
Integrated correlation analysis among gut microbiota, metabolites, and host metabolic markers. (**A**) Correlations between gut genera, SCFAs, bile acids and serum/liver indices; (**B**) Correlations between SCFAs, bile acids and genes. * *p* < 0.05, ** *p* < 0.01, *** *p* < 0.001.

Correlation analysis integrating host metabolic indices, gene expression, gut microbiota abundances, and fecal bile acid and SCFA levels revealed distinct associations among taxa, metabolites, gene expression, and host metabolic markers (Figure 6A,B). Genistein-enriched *Alloprevotella*, *Akkermansia*, and *Parabacteroides* were negatively associated with TC (*r*_s_ = −0.81, −0.85, −0.81, respectively; all *p* < 0.01), whereas *Dubosiella* showed a positive association with TC (*r*_s_ = 0.67, *p* < 0.05). For SCFAs, butyrate was positively correlated with genistein-enriched norank_f__Oscillospiraceae, Lachnospiraceae_NK4A136_group, *Alloprevotella*, and *Akkermansia* (*r*_s_ = 0.79, 0.71, 0.81, and 0.86, respectively; corresponding *p*-values were <0.01, <0.05, <0.01, and <0.01), but negatively correlated with high-cholesterol-diet-enriched *Faecalibaculum*, *Dubosiella*, *and Coriobacteriaceae*_UCG-002 (*r*_s_ = −0.77, −0.83, and −0.71, respectively; corresponding *p*-values were <0.05, <0.01, and <0.05, respectively). Propionate was positively associated with *Akkermansia* and *Bacteroides* (*r*_s_ = 0.84, 0.81, respectively; all *p* < 0.01), and acetate was positively associated with *Bacteroides* (*r*_s_ = 0.78, *p* < 0.05). For bile acids, UDCA was positively correlated with norank_f__Desulfovibrionaceae (*r*_s_ = 0.68, *p* < 0.05) and *Parabacteroides* (*r*_s_ = 0.98, *p* < 0.001). TαMCA was negatively correlated with *Dubosiella* (*r*_s_ = −0.53, *p* < 0.05) and positively correlated with *Alloprevotella* (*r*_s_ = 0.66, *p* < 0.05) whereas TβMCA was positively correlated with norank_f__Oscillospiraceae (*r*_s_ = 0.68, *p* < 0.05). UDCA, propionate, and butyrate were negatively associated with TC (*r*_s_ = −0.75, −0.66, −0.68, respectively; all *p* < 0.05). Correlation analysis of mRNA expression and host metabolic markers further showed that propionate, UDCA, and TαMCA were inversely associated with *Fxr* (*r*_s_ = −0.68, −0.90, and −0.66, respectively; corresponding *p*-values were <0.05, <0.01, and <0.05, respectively), whereas acetate, propionate, butyrate, and UDCA were positively correlated with *Cyp7a1* (*r*_s_ = 0.81, 0.72, 0.75, and 0.73, respectively; corresponding *p*-values were <0.01, <0.05, <0.05, and <0.05). UDCA, TβMCA, and butyrate were also positively correlated with *Abcg8* expression (*r*_s_ = 0.86, 0.71, and 0.71, respectively; corresponding *p*-values were <0.01, <0.05, and <0.05). Overall, these multi-omics correlation analyses revealed distinct associations among gut microbiota, microbial metabolites, gene expression, and host metabolic markers, suggesting that the cholesterol-lowering effect of genistein may involve coordinated changes in the gut microbiome and its derived metabolites.

## 4. Discussion

Hypercholesterolemia remains a major modifiable risk factor for cardiovascular disease worldwide, and dietary bioactive compounds may complement existing lipid-lowering strategies. In the present study, purified genistein (40 mg/kg body weight/day for eight consecutive weeks), isolated from fermented soybean meal and used as a defined bioactive isoflavone, significantly attenuated hypercholesterolemia in mice fed a high-cholesterol diet, as evidenced by reduced serum and liver TC. Our results are consistent with a recent study showing that genistein ameliorates hepatic cholesterol efflux [15]. A previous study also reported that genistein ameliorates metabolic fatty liver by modulating gut microbiota [21]. However, the mechanisms by which genistein alleviates hypercholesterolemia in relation to gut microbiota, SCFA, and BA metabolism remain to be fully elucidated. Therefore, the present study aimed to investigate the effects of genistein on high-cholesterol diet-induced hypercholesterolemia and to explore whether these effects were associated with changes in the gut microbiota–bile acid–SCFA axis.

Our results demonstrate that genistein reshaped the gut microbial community by reversing the high-cholesterol diet-induced reduction in alpha diversity and decreasing the Firmicutes/Bacteroidetes ratio, a signature positively associated with body mass and circulating cholesterol [22]. Genistein enriched several beneficial taxa, including *Akkermansia*, *Alloprevotella*, *Parabacteroides*, norank_f__Muribaculaceae, norank_f__Desulfovibrionaceae, and Lachnospiraceae_NK4A136_group. Human multi-omics evidence identifies A. muciniphila as a key cholesterol-lowering bacterium associated with cholesterol-to-coprostanol conversion and fecal sterol excretion [23]. The cholesterol-lowering effect of *A. muciniphila* is also linked to its promotion of bile acid metabolism [24]. These cholesterol-lowering functions of *Akkermansia* are consistent with its negative correlation with serum total cholesterol observed in the present study. Notably, norank_f__Muribaculaceae was significantly enriched by genistein and exhibited a negative correlation with serum TC in our correlation analysis. A previous study showed that norank_f__Muribaculaceae abundance was inversely associated with cholesterol levels [25]. In a study of *L. plantarum* H6-derived exopolysaccharide, norank_f__Muribaculaceae was also negatively correlated with multiple cholesterol-related parameters and was identified as a key taxon associated with cholesterol metabolism [26]. These observations are consistent with a negative association between norank_f__Muribaculaceae abundance and cholesterol levels. Collectively, the genistein-induced enrichment of *Akkermansia* and norank_f__Muribaculaceae, together with their negative correlations with serum cholesterol, suggests that these taxa may be associated with the hypocholesterolemic effect of genistein.

The microbial shifts induced by genistein were accompanied by significant alterations in intestinal bile acid profiles. Fecal UDCA content was elevated in the GEN group. UDCA is generally considered an intestinal FXR antagonist that reduces FGF15/19 production. Consistent with this mechanism, a randomized study in morbidly obese patients with non-alcoholic fatty liver disease showed that short-term UDCA administration reduced circulating FGF19 and FXR activation [27]. However, a recent study reported that UDCA acted as an ileal FXR agonist in male mice with hepatic FXR deficiency [28]. This discrepancy may reflect context-dependent effects related to tissue-specific FXR status and the competition from other endogenous bile acid ligands. In the present study, the elevation of fecal UDCA was accompanied by reduced ileal *Fxr* and *Fgf15* mRNA expression, suggesting potential suppression of FXR–FGF15 signaling, consistent with its antagonistic properties on intestinal FXR. More pronounced changes were observed for TαMCA and TβMCA, which were increased by 234.38% and 301.76%, respectively. Both are murine-specific primary bile acids and competitive, reversible FXR antagonists with IC50 values of 28 μM and 40 μM, respectively [29]. Critically, TβMCA-mediated FXR inhibition is intestine-specific, with no significant inhibition of hepatic FXR signaling [29,30]. TαMCA and TβMCA positively correlated with genistein-enriched Alloprevotella and norank_f__Oscillospiraceae but negatively with *Coriobacteriaceae_UCG-002* and *Faecalibaculum*, suggesting that genistein may increase these FXR-antagonistic bile acids, potentially through microbial modulation.

In parallel, genistein treatment robustly elevated fecal butyrate and valerate levels, with propionate restored to levels comparable to normal control groups. SCFAs have been reported to exert cholesterol-lowering effects through multiple mechanisms: butyrate upregulates hepatic CYP7A1 expression, the rate-limiting enzyme in bile acid synthesis, thereby accelerating the conversion of cholesterol into bile acids and promoting fecal bile acid excretion [31]; propionate inhibits hepatic cholesterol synthesis from acetate and mevalonate in hepatocytes [32]; and SCFAs also modulate intestinal cholesterol absorption by upregulating ABCG5/ABCG8 expression, which promotes cholesterol efflux from enterocytes back into the intestinal lumen for excretion [33]. Additionally, SCFAs have been shown to act as indirect antagonists of FXR signaling, thereby reducing enterohepatic circulation and regulating cholesterol metabolism [31]. In the present study, the genistein-induced increases in butyrate and propionate, together with the elevated TαMCA/TβMCA levels, may contribute to potential suppression of FXR signaling, thereby contributing to the observed increase in hepatic *Cyp7a1* mRNA expression and suggesting enhanced bile acid synthesis. Butyrate was positively correlated with genistein-enriched *Akkermansia*, *Alloprevotella*, and Lachnospiraceae_NK4A136_group, while propionate was positively associated with Akkermansia and Bacteroides.

The molecular mechanism underlying the cholesterol-lowering effect of genistein may involve modulation of the enterohepatic FXR–FGF15 axis. In our study, genistein administration was associated with reduced ileal mRNA expression of *Fxr* and the FXR-related genes *Fgf15*, *Ibabp*, and *Asbt*. The FXR–FGF15 axis is a key negative feedback regulator of bile acid synthesis: intestinal FXR induces FGF15, which represses hepatic CYP7A1 [34], with intestinal rather than hepatic FXR activation mediating short-term CYP7A1 suppression [35]. Since CYP7A1 is the rate-limiting enzyme converting cholesterol to bile acids [36], relieving this inhibition promotes cholesterol conversion. Consistent with this possibility, we observed elevated hepatic *Cyp7a1* mRNA expression in genistein-treated mice. The concurrent increases in hepatic *Abcg5*, *Abcg8*, and *Abca1* mRNA expression may suggest enhanced cholesterol efflux into bile and the intestinal lumen, as ABCG5/G8 functions as a heterodimer that promotes the secretion of cholesterol and xenosterols into the bile and intestinal lumen [37]. Furthermore, the reduced mRNA expression of *Srebp2* and *Hmgcr* suggests that genistein may reduce endogenous cholesterol synthesis at the transcriptional level, as SREBP2 is a major transcriptional regulator controlling the expression of cholesterol synthesis genes such as HMGCR [38].

## 5. Limitations

Several limitations should be acknowledged. First, this study was conducted in a mouse model of diet-induced hypercholesterolemia; therefore, the findings cannot be directly extrapolated to humans, and clinical validation is needed before any human application. Second, purified genistein was used rather than fermented soybean meal itself; thus, the results should not be directly generalized to fermented soybean meal or its crude extracts. The present findings should be interpreted as mechanistic evidence rather than as a nutritional recommendation for fermented soybean meal or soy foods. Third, the mechanistic conclusions are based mainly on mRNA expression and correlation analyses. Protein-level and functional validation, as well as causal experiments such as fecal microbiota transplantation, bacterial colonization, and metabolite supplementation, are needed to confirm the proposed mechanisms. Fourth, the sample size was modest (n = 5–6 per group), and the microbiome and metabolomics analyses were exploratory. Future studies with larger sample sizes in animal models and, ultimately, clinical trials are needed to validate these findings. Finally, the study was relatively short-term, and the genistein dose (40 mg/kg/day) was selected for mechanistic investigation and is higher than typical dietary isoflavone intake (0.25–0.83 mg/kg/day in Asian populations and <0.03 mg/kg/day in Western populations). After body surface area normalization, this dose corresponds to a human equivalent dose of 3.24 mg/kg/day, which is within the range used in human supplementation trials and below the 50 mg/kg/day no observed adverse effect level from a 52-week rat study [39]; however, the long-term efficacy and safety of genistein at pharmacologically active doses remain to be determined.

## 6. Conclusions

Genistein effectively ameliorated diet-induced hypercholesterolemia and body weight gain in mice. The underlying mechanism may involve the modulation of bile acid- and SCFA-producing gut microbiota and the potential suppression of the intestinal FXR–FGF15 pathway, which may collectively contribute to the maintenance of cholesterol homeostasis. These findings provide potential mechanistic insights into genistein as a promising dietary bioactive compound for the management of hypercholesterolemia and may support the development of genistein-based functional ingredients from soybean-derived materials.

## Data Availability

The original contributions presented in this study are included in the article/Supplementary Materials. Further inquiries can be directed to the corresponding authors.

## Supplementary Materials

The following supporting information can be downloaded at: https://www.mdpi.com/article/10.3390/foods15213986/s1, Figure S1: TLC and HPLC of genistein obtained; Table S1: Composition of the normal and high-cholesterol diet; Table S2: List of primers used in this study.
