# Supplementary material for: Genistein Alleviates Diet-Induced Hypercholesterolemia via the Gut Microbiota–Bile Acid–SCFA Axis in Mice
Source: Foods. 2026 Nov 6;15(21):3986. doi: 10.3390/foods15213986 (PMC13763354; doi:10.3390/foods15213986)
Supplement: Supplementary file 1 [file foods-15-03986-s001.zip › foods-4597937-supplementary.pdf]

Supplemental files

Figure S1 TLC and HPLC of genistein obtained

b

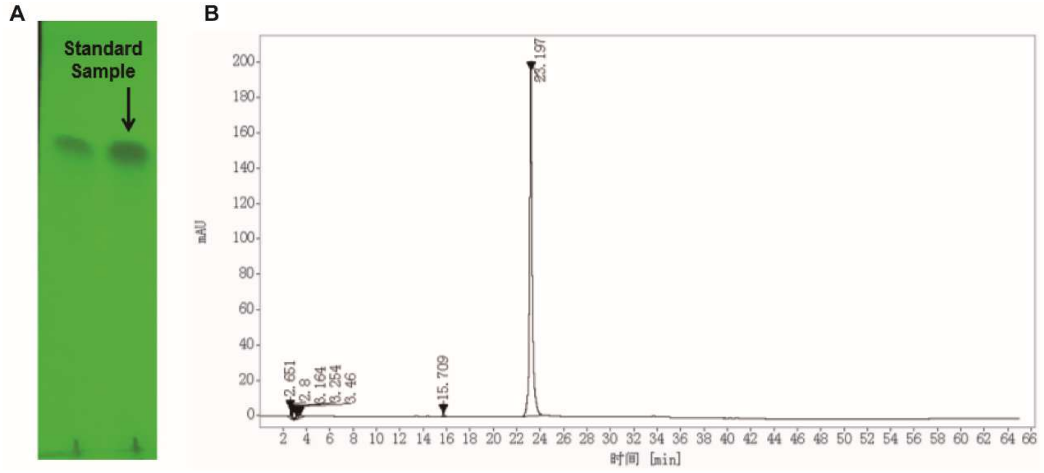

Figure S1TLC and HPLC of genistein obtained (A) TLC of genistein after purification with HPLC. (B) HPLC of genistein obtained

Table S1. Composition of the normal and high-cholesterol diet.

Ingredient	Normal diet	High-cholesterol diet
Corn starch	472.1g	472.1g
Sucrose	110.3g	110.3g
Lard	64.8g	64.8g
Casein	223.8g	223.8g
Gelatin	18.9g	18.9g
Mineral Mix	40.0g	40.0g
Vitamin Mix	20.0g	20.0g
Cellulose	50.0g	50.0g
Cholesterol	0	12.7g
Bile salt	0	5.1g
Total	1000.0 g	1017.8 g

Table S2. List of primers used in this study

Genes	Primers	Primers sequence
Fxr	Forward	5'- ACATCCCCATCTCTCTGCAC -3'
	Reverse	5'- TGTGAGGGCTGCAAAGGTTT -3'
Ffg15	Forward	5'- ACGTCCTTGATGGCAATCG -3'
	Reverse	5'- GAGGACCAAAACGAACGAAATT -3'
I-babp	Forward	5'- CTTCCAGGAGACGTGATTGAAA -3'
	Reverse	5'- CCTCCGAAGTCTGGTGATAGTTG -3'
Asbt	Forward	5'- GTCTGTCCCCCAAATGCAACT -3'
	Reverse	5'- CACCCCATAGAAAACATCACCA -3'
Cyp7a1	Forward	5'- GGGATTGCTGTGGTAGTGAGC -3'
	Reverse	5'- GGTATGGAATCAACCCGTTGTC -3'
Cyp8b1	Forward	5'- AAGGCTGGCTTCCTGAGCTT -3'
	Reverse	5'- AACAGCTCATCGGCCTCATC -3'
Srebp2	Forward	5'- CAGGTGCAGACGGTACAGG -3'
	Reverse	5'- CGACCCTTACTGGCACTTGAA -3'
Hmgcr	Forward	5'- AGCTTGCCCGAATTGTATGTG -3'
	Reverse	5'- TCTGTTGTGAACCATGTGACTTC -3'
Abcg5	Forward	5'- AGCTCTTCCAACACTTCGAC -3'
	Reverse	5- TACGTTTCTATTTCCCGCTC -3'
Abcg8	Forward	5'- GGCTCAGGATCGGCTTTCAC -3'
	Reverse	5'- CCTTGACACAGGCATGAAGCA -3'
Abca1	Forward	5'- TTGGCGCTCAACTTTTACGAA-3'
	Reverse	5'- GAGCGAATGTCCTTCCCCA-3'
β-actin	Forward	5'- AAGTCCCTCACCCTCCCAAAAG -3'
	Reverse	5'- AAGCAATGCTGTCACCTTCCC -3'
